# Mobile learning devices in the workplace: ‘as much a part of the junior doctors’ kit as a stethoscope’?

**DOI:** 10.1186/s12909-016-0732-z

**Published:** 2016-08-17

**Authors:** Rebecca Dimond, Alison Bullock, Joseph Lovatt, Mark Stacey

**Affiliations:** Rebecca Dimond, Cardiff School of Social Sciences, 10/12 Museum Place, Cardiff, CF10 3BG UK

**Keywords:** Technology enhanced learning, Smartphones, Workplace learning, Patient care, Learning resources, Quality improvement

## Abstract

**Background:**

Smartphones are ubiquitous and commonly used as a learning and information resource. They have potential to revolutionize medical education and medical practice. The iDoc project provides a medical textbook smartphone app to newly-qualified doctors working in Wales. The project was designed to assist doctors in their transition from medical school to workplace, a period associated with high levels of cognitive demand and stress.

**Methods:**

Newly qualified doctors submitted case reports (*n* = 293) which detail specific instances of how the textbook app was used. Case reports were submitted via a structured online form (using Bristol Online Surveys - BOS) which gave participants headings to elicit a description of: the setting/context; the problem/issue addressed; what happened; any obstacles involved; and their reflections on the event. Case reports were categorised by the purpose of use, and by elements of the quality improvement framework (IoM 2001). They were then analysed thematically to identify challenges of use.

**Results:**

Analysis of the case reports revealed how smartphones are a viable tool to address clinical questions and support mobile learning. They contribute to novice doctors’ provision of safe, effective, timely, efficient and patient-centred care. The case reports also revealed considerable challenges for doctors using mobile technology within the workplace. Participants reported concern that using a mobile phone in front of patients and staff might appear unprofessional.

**Conclusion:**

Mobile phones blur boundaries between the public and private, and the personal and professional. In contrast to using a mobile as a communication device, using a smartphone as an information resource in the workplace requires different rituals. Uncertain etiquette of mobile use may reduce the capacity of smartphone technology to improve the learning experience of newly qualified doctors.

## Background

Seeking information is a critical skill within medicine, yet how information is acquired and applied by newly qualified doctors, remains widely unexplored within sociological and medical education literature. New modes of information delivery have moved information seeking from the ‘back-stage’ of libraries and offices to the ‘front-stage’ of clinical encounters. Smartphone technology offers doctors the opportunity to access up-to-date, reliable information at the bedside. While developments in information technology have contributed to an information explosion, they have also provided dynamic modes of information delivery. The ever-increasing information needs of doctors and the considerable difficulties of keeping personal stocks of knowledge up-to-date was noted almost 20 years ago [1]. With the introduction of smartphone technology, a mobile phone has become a viable tool to address clinical questions and mobile learning holds the potential to revolutionize medical education and medical practice, supporting the transition from novice to expert [2].

Much has been written about the distinction between novice and expert [3]. Distinguishing characteristics include a greater base of knowledge which experts can quickly recall and deploy and effective automated procedures which lessen the burden on working memory [4]. Clark and Clark [5] explain how cognitive load is affected by the amount of automated prior knowledge: less prior knowledge means more mental effort is needed to perform the task, and “the more automated the prior knowledge, the less cognitive effort required” (p209). Although this paper is concerned with the ready access to explicit or declarative knowledge, rather than automated (or implicit, procedural) knowledge, novice doctors depend on this as a stage in their development of automaticity over time. Further, by enhancing access to knowledge, smartphone technology may support an immediate benefit in lessening the load on working memory.

The use of mobile resources within medicine appears widespread, with many studies reporting positive attitudes of health professionals and medical students [6–8] and calls to encourage and support greater use [6, 9, 10]. However, smartphones also attract apprehension, reflecting continuing fears about the use of new technology to access medical information [11] with appeals for more stringent policies [12, 13]. Concerns have been expressed about dependency on technology and its substitution for clinical thinking [14], the potential for distraction [12] and the conceivable threat to patient privacy due to photographic facility [15]. There are considerable barriers to using smartphones to access textbooks within a learning environment, including problems of synchronisation with alternative resources and the cost, not only in terms of producing text books in a ‘mobile’ format, but also the cost of personal smartphone ownership [6].

With an ever increasing number of apps available, there is also the question of the reliability of an information source [16]. The blurring of the boundary between apps and ‘plug-ins’ (hardware extras which convert the smartphone into a medical device such as thermometer, blood pressure or heart rate monitor) raises regulation issues. Medical devices used in the diagnosis or treatment of patients need to be registered with the Medicines and Healthcare Products Regulatory Authority (MHRA) in the UK or comply with the Food and Drug Administration (FDA) in the US or the CE mark in Europe [17, 18]. The iDoc Project remains one of the few studies which aims to consider how smartphones are used within medicine to support workplace learning. Our focus is the ‘foundation’ years of medical training in the UK, which are recognised as a period of transition following medical school to workplace, associated with high levels of cognitive demand and stress [19], and increased patient mortality [20]. At a time when doctors need to gain the skills and confidence to work without supervision, this transition can be supported by providing rapid access to reliable information via a smartphone [21].

After reporting evidence that demonstrates how the textbook app supports new doctors in their provision of safe and efficient patient care, we explore challenges arising from the social rituals and expectations associated with smartphone use. Our research questions are twofold: Does usage of a mobile textbook app (a) support newly qualified doctors in providing safe and efficient patient care and (b) present boundary challenges to the doctors’ workplace practice?

## Methods

The aim of the iDoc Project is to support newly qualified doctors by providing internet-free access to reliable information on a mobile device. The iDoc Project is a collaboration between a postgraduate educational organisation (Wales Deanery) who fund the textbook smartphone app for all newly qualified doctors working in Wales, and the researchers based at Cardiff University School of Social Sciences who evaluate its use. When the project was initiated (2009–2010) newly qualified doctors were offered personal digital assistants (PDAs) preloaded with text books, and in the second phase (2010–2011) smartphones were offered. Following feedback from participants about the problems of carrying two devices (the project phone and their own, often more up-to-date device), participants were provided with a licence key to download the text books, via an app, onto their own device (Phase 3).

This article draws on data and evaluation from Phase 3 of the iDoc Project (August 2012 – July 2013). Newly qualified doctors (foundation years one -F1- and two -F2) throughout Wales were offered Dr Companion© software with access to five key medical texts (the British National Formulary (BNF), the Oxford Handbook of Clinical Medicine, the Oxford Handbook of Emergency Medicine, the Oxford Handbook of Clinical Specialities and the Oxford Handbook of Clinical Surgery). The selection of these books was led by author, clinician and medical educator Mark Stacey and informed by feedback from trainees from early phases of the project. Unlike e-books, these app books are specifically formatted for use on mobile devices and the software includes a cross-search facility. Participation in the project was optional and in phase 3 overall uptake was 58 % (374 out of a total of 643 foundation doctors), an increase on earlier years. The sample comprised 70 % F1s and 60 % females.

Completion of a baseline questionnaire was required before a licence was allocated. This collected data on frequency, type, perceived value and variation in use of workplace information sources. At the end of the year participants completed an exit questionnaire. The analysis of the questionnaire data has been published previously [22]. During the year, participants were asked to submit narrative case reports which detailed use of the mobile resource in action. Participants were encouraged to submit at least two case reports, although this remained voluntary and there were no penalties for users who did not submit case reports. The case reports were submitted via a structured online form (using Bristol Online Survey - BOS) which gave participants headings to elicit a description of: the setting/context; the problem/issue addressed; what happened; any obstacles involved; and their reflections on the event. Participants were asked to describe what they would do if they did not have the app on their device. Collected data were confidential but not anonymous, and all questions were open and optional. To encourage participants to submit case reports, word length was unrestricted, leading to variable accounts (word length ranged from 33–996) although most were between 250 and 450 words. Of those who submitted case reports, 37 % submitted one case report, 60 % submitted two and 4 % submitted three or more. Data reported here are from these phase 3 case reports (*n* = 293). Research ethics approval for the iDoc evaluation was obtained from Cardiff University (02/12/2010). Consent was taken from participants as part of the on-line sign up process.

The substantive content of case reports was analysed thematically in three main stages. Two authors (RD and JL) independently read all the reports and highlighted key themes which were discussed and agreed with AB and MS. The reports were then read and categorised by the purpose of use by two authors (RD and JL) and these were discussed with AB and MS. Then RD, JL and AB re-categorised the reports using the elements of the quality improvement framework (IoM 2001) [23] relating to safety, effectiveness, timeliness, efficiency and patient-centredness [see Table 1]. The classification was reviewed by MS and agreed by all members of the research team.

**Table 1:** Thematic scheme with examples

Theme	Example
Safe care	Information seeking
	- simple: checking medicine dosages
	- complex: management of patients with rare/unknown conditions
Effective care	Making evidenced-based decisions
Timely	Quickly accessible information resource
	Timely patient management and treatment
Efficiency	Identifying appropriate and relevant investigations
	Supporting rapid decision-making
Patient-centred	Sharing information

## Results

### Use of the smartphone app to support newly qualified doctors

The quality improvement framework [23] provides a mechanism for drawing attention to the important ways that the textbook app was used in practice to support the work of newly qualified doctors. To present an overview of our data, we report findings under the framework headings, recognising that these elements overlap.

### Safe care

Many of the case reports referred to the use of the app for basic information seeking, for example, checking medicine dosages. The doctors described using the app to confirm dosages when they were not completely sure: ‘The iDoc app is often of most use when there is a situation where I am pretty confident I know what to do, but not 100 % certain; this helps to reassure and confirm my actions’ (F1 #5). Trainees specifically used the word ‘safe’ or ‘safety’ when describing the app’s facility to check medications and dosage: ‘[It’s] really useful as it is quick and safe’(F1#11); ‘It takes a few seconds to double check a dose for example, it is improving patient safety, especially during busy ward rounds’ (F1 #136).

Using the app to enhance patient safety extended beyond simple medication checks. Others referred to the textbook app to support more complex problems:

I had not come across a clinical case of hypothermia and was uncertain how to manage this patient. I was able to look up the over-view of hypothermia and initiate the appropriate management. I was guided by iDoc to start rewarming the patient slowly and to monitor her cardiac activity for arrhythmias. (F1 #26)

In such circumstances, use of the app can support decision-making, meaning treatments can be speedily implemented to the benefit of patient care.

### Effective and appropriate care

Effective care is evidence-based and employs the best available methods. The app supported the trainee doctors’ decision-making in managing complex problems, particularly when they have little prior experience:

Whilst reviewing blood results I noted hypercalcaemia in a patient who had been admitted with shortness of breath and a tender left scapula … I was familiar with hypercalcaemia management, but … I wanted to check I hadn't missed anything. Using the application to access the Oxford Handbook of Clinical Medicine I was reminded myeloma was a cause of hypercalcaemia. As a result I requested [tests] … Once again I checked the iDoc app and was reminded to request skeletal survey. … The information meant investigations were done quicker and meant that by the time specialists were reviewing the patient they had more evidence to aid their opinion. [F1 #22]

This extract demonstrates how newly qualified doctors used the app to call for appropriate investigations and ensure their decisions were evidence-based. Many of the case reports identified the specific resources used, the extract #22 for example, refers to the Oxford Handbook of Clinical Medicine. The set of books on the app are up-to-date and provide the information needed to inform novice doctors’ diagnosis and treatment planning.

### Timely

The provision of timely attention and reduction in waiting time is of course a key concern within medicine. Some extracts above have already pointed out time saving benefits (particularly #22). At a basic level, having information to hand could save doctors’ time in searching for books on the ward, waiting to access the internet or awaiting the availability of a senior colleague: ‘If I did not have the app I would have to use the internet or find a BNF. This can be difficult in a busy A&E [accident and emergency] department where it’s difficult to find BNF or get access to computers’ (F1 #123). One succinctly stated that ‘accessibility and reliability of information anywhere in the workplace saves tremendous amounts of time every day’ (F1 #77). Other case reports show how use of the app has reduced delays in patient management and treatment:

Child had been unwell with tonsillitis, on antibiotics. Initially improving then developed non blanching rash worse over groin and thighs. I thought this may be HSP [Henoch-Schonlein Purpura] but having never seen it [I] wasn’t entirely sure. Needed correct diagnosis to further manage child. Used the iDoc app to correctly diagnosis patient with HSP using information and photos from clinical specialities … If I didn’t have iDoc I would have had to use the Internet or refer to paediatrics without a definite diagnosis. It would have resulted in a delay in patient care and management. (F2 #291)

The reports reveal the hidden labour of seeking information: it takes time and effort. The doctors described how using a mobile resource saved time, allowing the doctor to ‘manage the patient’s condition quickly and efficiently’ (F2 #281). Likewise, wasting time was also prominent, as this means time away from addressing the needs of patients. Going off the ward to search for text books, or waiting for colleagues to return from theatre before asking for advice, all have implications for the speed with which patients are managed.

### Efficiency

Efficiency involves seeking to reduce waste of supplies, equipment, space, capital, ideas, time and energy within the healthcare system. A principal way in which the textbook app supports efficient care is how trainee doctors use it to determine appropriate and relevant investigations, rather than calling for an unnecessary array of tests.

A patient with a known pituitary lesion was admitted under our care. She had presented generally unwell due to a likely urinary tract infection [UTI], and had gradually become less responsive over days … I was asked by my consultant to refer to resources on my iPhone to determine which tests would be needed … We discussed the case as a team with the aid of the OHCM [Oxford Handbook of Clinical Medicine] and set about requesting relevant lab tests. [Without the app] it would have taken much longer to ensure the relevant tests were performed … We also felt that we were not requesting irrelevant tests or receiving too many confusing or conflicting test results all at the same time. … (F1 #76)

### Patient-centred

Patient-centred care, which is respectful of and responsive to individual patient preferences, cultural values, social context, and specific needs, is reflected in many current healthcare policies (see for example Department of Health (UK)) [24]. The following extract from the case reports describes the new doctor using the app in front of patients and sharing the results:

Many of the usual treatments for endometriosis were inappropriate as [patient] was keen to conceive. With the agreement of the consultant we agreed to continue managing her symptoms conservatively, but the patient was concerned as I could not tell her from memory how safe these drugs were in pregnancy … I used iDoc to look up the information I needed and physically show it to the patient which I felt reassured her. (F2 #241)

Although involving the patient in this way may only serve to ‘reassure’, this instance highlights the possibility of actively involving the patient in shared decision-making. Showing the patients what the doctor was looking at might not seem ground breaking, however, this practice was uncommon. Significantly, phone use in front of patients and colleagues was sometimes viewed as problematic. In a later section we explore how the use of mobile phones within a healthcare environment can present boundary challenges.

### Supporting the novice learner in a challenging environment

The quality improvement framework is useful to indicate the various factors that make up good quality medical care. But what many of the case reports also revealed was how the textbook app was particularly useful because the doctors were novices. Many of the case reports referred to the new doctors being in new and potentially stressful situations, where they needed to make the right decisions for both the patients and their senior supervisors. Several, for example, referred to being on night shift or on call for the first time, or working on their own. These were situations with high cognitive load and as novices they had limited automated knowledge [5]. Having ready access to up-to-date information allowed the doctors to feel confident in their work and provided the knowledge to confirm that they were working correctly. This was the case for one trainee who stated:

[I had] limited surgical experience at the time (first on-call shift). [I] wished to consider all options prior to presenting to seniors … The app enabled me to feel more confident with my provisional diagnosis (F1 #89)

In the following extract the new doctor describes how he/she used the app ‘on the way to the ward’ to prepare for an examination of the patient and importantly, *before* discussion with a senior colleague:

I was called to the ward to see a patient who was scoring highly on his MEWS [Modified Early Warning Score] chart during one of my first medical twilight shifts. I wanted to refresh myself on what to do in this situation and make sure I covered everything I needed to. I was able to look on iDoc on my way to the ward so I didn’t waste any time in getting to the unwell patient. When I arrived I knew exactly what to do and was able to start examining and requesting appropriate investigations before … I phoned my senior for help. (F1 #80)

Many of the case reports specifically noted that the app was a useful tool that supported both personal and collective learning. One trainee doctor (F1 #63) spoke of actively using the app ‘with other colleagues and it helps to stimulate ideas and discussion, that ultimately lead to improved care’. In another case report (F2 #279) the trainee described how he/she used the app with colleagues ‘to see if we could generate any more ideas and to gain further understanding of the ideas we had already come up with’.

### Uncertainty of use

Despite the evident beneficial role that mobile resources play in supporting newly qualified doctors, it is possibly not surprising that new technology can also present challenges. Specifically, some participants expressed concern about how or whether to use their phone in front of patients and ward staff. Mobile phone use presents a visible challenge to tacit rules of ward behaviour. The case reports reveal that many of the doctors were aware that using their mobile phone contests workplace boundaries, with the potential to attract unwelcome attention and criticism. Uncertainty of use was related to concerns about how others might misconstrue their use of a mobile phone and judgements were made about the extent to which colleagues or patients might be open to the use of new technology. This was the case when referring to older patients for example, who were characterised as lacking awareness of smartphones and therefore potentially reluctant to accept their use to support mobile learning:

I did not want to use the application in front of an elderly patient as I think it would have looked unprofessional, certainly with the older population who may be less aware of the clinical use of such technology. (F1 #47)

I still feel awkward if people (staff or patients) see me using my phone at work so I only ever use it when there is no--one else around or if I can go somewhere more private to look things up. (F2 #263)

One of the main reasons why there is uncertainty over using a mobile phone and concern about the image it might project is because of the blurring between work and leisure activities. In particular, mobile phones can be used for personal leisure activities such as social networking or playing games, and this was suggested as the reason why their use may be frowned upon by colleagues:

Sisters/nurses on the ward have asked me to put my phone away in a clinical area. Even when [I] explained that I am not using it for personal reasons, it can be difficult to use it in this situation. (F2 #236)

Nurses thought that I was checking my text messages (F1 #179)

Newly qualified doctors were acutely aware that using a mobile phone might threaten their professional image. Indeed, many of the case reports highlight how use of a phone can attract attention from other members of staff:

On several occasions I have found that when using the app on my phone, nursing staff approach me with various jobs as they think that I am not busy with work (despite informing them that I am using the app). I find it difficult to use the app whilst on the ward as it looks as though I’m texting and not doing work related jobs. (F2 #219)

The case reports revealed that the doctors developed personal strategies to address uncertainty over mobile use. For example, as with the previous example (#47) judgements were made about when it might be appropriate to use a mobile phone, and when not. Some new doctors chose not to use their phone in front of others, attempting to find somewhere ‘more private’ (F2 #263) or ‘away from the clinical case’ (F2 #242) for example. Using their phone away from other people in order to avoid criticism of unprofessional behaviour suggests barriers to the point-of-care potential for mobile resources being realised in practice.

Some doctors found that they could combat potential criticism by being overt about the resources they were accessing, although as F2 #219 suggested, this was not always successful. The strategy employed was to offer a clear explanation as to why they were using their mobile phone:

Some of the members of staff see you using your phone and look disapprovingly at you but once you explain what you are doing they were happy with me using my phone. (F1 #105)

As the patient was an elderly gentleman I was slightly apprehensive that he wouldn’t appreciate me using a phone during the consultation however with explanation of my actions he was perfectly content with my use of iDoc. (F2 #240)

The newly qualified doctors recognised that the way they were using their phone was important. By sharing the resources with patients, their mobile phone facilitates patient centred care but it also avoided the potential for criticism and uncertainty. Providing explanation, to patients or ward staff, as to why a mobile phone was being used within a work space usually enabled the doctors to proceed with confidence. Rather than being secretive about their mobile use by moving away from the patient or nurses station, the strategy was to become more visible. A previous extract (F2#241) has highlighted how the screen can be shown to patients so that they are aware of what information the doctor is using. For one trainee doctor, resting the phone on a table ensured that anyone watching would be able to see the screen, recognise how the user was interacting with the device and would therefore not question its use:

I tend to use it whilst it’s resting on the table so that anyone glancing at the phone can immediately see that it is text and not a game! (F2 #226)

Through openness, including education and increased visibility, the doctors were able to re-position their mobile phone as a work tool, and its use as legitimate. Part of the perceived problem was a lack of awareness of how a mobile phone has become an essential tool in the ‘doctors’ kit’:

Patients and staff probably have yet to come to terms with the fact that the smartphone is now as much a part of the junior doctors’ kit as a stethoscope. (F1 #179)

A significant barrier to greater use of the resource is that a range of actors, including patients, ward staff, senior medical colleagues, as well as hospital managers, policy makers and medical educators, have yet to demonstrate their trust in newly qualified doctors using mobile technology appropriately.

## Discussion

We began by asserting that a smartphone is a valuable tool for a newly qualified doctor. The development of smartphone technology, particularly in terms of portability and multi-functionality, has brought into sharp focus the possibilities of information seeking at the ‘point-of-care’, particularly in time-critical situations [25]. Smartphones are unrivalled as an information resource - these devices are light and portable and because of personal ownership, easily accessible. This contrasts with more traditional resources such as textbooks which are often out-of-date, heavy to carry or hard to find; ward-based computers which can be difficult to locate or are occupied by others and senior staff who might be busy and might see minor questions as disruptive. However, like Aubusson et al. [26] we also highlight how even within the context of a professional working and learning environment, social and cultural meanings associated with mobile technology remain significant.

The social consequences of mobile phones are well documented. The speed of development of mobile technologies and the power of the mobile phone to influence the social arena has led to its identification as a cultural icon of the 21st century [27]. However, as highlighted by Agger [28] ‘iTime’ can also be associated with a degree of powerlessness, where ‘smartphones use us, bending us to their compulsive rhythms and demanding our attention’ (p.119). Indeed, the power of the smartphone to influence and disrupt social relations, particularly within a learning environment, has been noted [12, 29].

Mobile devices complicate our understandings of place and space, facilitating both ‘absent presence’ and ‘isolated connectivity’ [30]. As communication devices, mobile phones have been lauded because they let people be ‘distant yet close’ [31], yet also criticised for allowing users to be ‘mentally and socially elsewhere’ [32] thus creating a conflict with the norms of expected behaviour. Drawing on Goffman’s [33] rules of social interaction, Ling [27] highlights how talking on a phone in public forces others to become an ‘audience’, often drawn unwittingly into the conversation. He suggests how this conflict was managed by mobile phone users, for example, by creating a ‘symbolic fence’ such as turning away from others or talking in hushed tones. However, when using a smartphone as a resource within a hospital environment, some trainee doctors appear to do the opposite, by attempting to make their use overt, transparent and public. The new doctors in our study highlight how they make considerable attempts to identify and engage their audience in an effort to dispel potential criticism. Whereas some might choose to move away from the patient, others will go to great efforts to tell people what they are doing, share images from the app or leave their phone open for anyone passing to see. This suggests new possibilities for patient engagement.

A smartphone is a multi-functional tool to support medical education and practice but it also presents challenges. How to use a ‘personal’ device within a ‘public’ professional workspace remains a challenge for newly qualified doctors [34], and here it has been important to show how the trainee doctors describe their own use of their mobile phone since ‘no technology ever speaks for itself’ [35]. Put simply, our data suggest that just because a resource has the potential to support mobile learning, it does not mean that it will be used for that purpose or have that desired outcome. The introduction of any new technology brings with it the potential for new uncertainties and opens up the space for both innovation and resistance. What is clear from our analysis is that the use of a smartphone as a work device requires negotiation. It has yet to achieve the unambiguous status of a stethoscope.

Smartphone resources are introduced within existing frameworks of policy and practice, where social encounters and the rules of behaviour are ritualised [33]. Using a mobile as a resource brings with it new rules of behaviour and also reveals ‘hidden’ expectations, particularly when the rules are challenged or broken. Many hospitals continue to regulate the use of mobile phones through formal policies, restricting their use within certain parts of the hospital or within departments. Our study also reveals the implementation of informal policies, where ward staff maintain surveillance to prevent mobile use. Advertising that doctors are using their mobile phones as an information resource might be useful in this case, to enhance both patient and staff awareness. Although it is outside of the remit for this project, it would be informative to examine how hospital regimes, for example, the use of iPad records at the end of patient beds could support smartphone use and enable greater integration.

We have a lot more to learn about how mobile technologies are used in practice, and how rules are decided and enacted within the hospital environment. Indeed our lack of understanding of smartphone use and associated etiquette is partly a reflection of the discrepancy between the speed of development in technology and the much slower academic model of research and dissemination. Breaches in expectations of behaviour, such as glancing at a phone from time to time, or it ringing on inappropriate occasions, can be collectively tolerated [27]. However, there is a lot at stake for doctors who violate social and professional norms. At the moment, we have a complicated picture where the rules of information seeking at the bedside are being constantly negotiated. Indeed, it will be important that smartphone use becomes not just tolerated but socially accepted and ritualised within the medical setting, if the possibilities of information technology are ever to be realised in practice.

## Conclusion

In the rapidly changing landscape of information technology, ‘what works’ in medical education can be difficult to answer. This study has several limitations, highlighting the practical aspects of introducing new mobile learning resources and how to evaluate them. Despite the large number of participants who were offered licence keys, the overall uptake of the app was fairly low at around 58 % of all newly qualified doctors in Wales (although it is noted that this rate was higher than in previous phases). Additionally, we know very little about the information seeking practices and challenges of those who chose not to use the textbook app or those who did not submit case reports. A more stringent approach has been put in place for later phases, where access to the app will only continue if case reports are submitted and data tracking is being used to identify those using the app and those who are not. It will be interesting to note whether such surveillance practices influence information seeking behaviour. Another major limitation is that users who did not submit their case reports were not penalised, and therefore, there was little incentive for them to comply with requests from the evaluation team. We also did not stipulate that all fields had to be filled in, which meant that some data were missing. This of course makes a difference in evaluation, and highlights the tension that educational researchers experience between supporting education and training on the one hand, and ensuring that data collection is adequately robust on the other.

Despite these limitations, we have extensive data to suggest how a mobile app which enables access to key medical textbooks can support newly qualified, novice doctors. By supporting effective, evidence-based decisions and reducing delays, use of the app inspired confidence in the newly qualified doctors. Yet in some cases, the social rituals surrounding mobiles impeded their use. This study therefore highlights that users of technology need technical skills, but also practical knowledge about how to use the tools appropriately. Newly qualified doctors might be ‘digital natives’ [36], yet we know little about how they gain tacit knowledge of how to integrate mobile resources with patient care and questions remain about how mobile etiquette is produced in medical spaces. It might be significant for the users of the resource, for example, that the app is only offered to F1s and F2s, and not their seniors. How to adapt to the changing pace of technology is an important question for educators [37] and future research needs to address the critical question of how to explore the social implications of technology within an ever changing environment. The rapid development of smartphone technology has meant that research surrounding mobile phone use already appears outdated. We encourage those who are introducing new technologies in medical education and professional practice to share their experiences, and for rapid publication of research results.
